# Supplementary material for: Model Approach to Thermal Conductivity in Hybrid Graphene–Polymer Nanocomposites
Source: Molecules. 2023 Oct 30;28(21):7343. doi: 10.3390/molecules28217343 (PMC10647783; doi:10.3390/molecules28217343)
Supplement: Supplementary file 1 [file molecules-28-07343-s001.zip › molecules-2622497-supplementary.pdf]

Model Approach to Thermal Conductivity in Hybrid Graphene–Polymer Nanocomposites

Andriy B. Nadtochiy [1], Alla M. Gorb [1], Borys M. Gorelov [2], Oleksiy I. Polovina [1], Oleg Korotchenkov [1,3] and Viktor Schlosser [4,*]

[1] Faculty of Physics, Taras Shevchenko National University of Kyiv, 01601 Kyiv, Ukraine; nadtku@univ.kiev.ua (A.B.N.); alla.gorb@knu.ua (A.M.G.); fantality@ukr.net (O.I.P.); olegkorotchenkov@knu.ua (O.K.)
[2] Chuiko Institute of Surface Chemistry, NAS of Ukraine, 17 General Naumov Str., 03164 Kyiv, Ukraine; bgorel@ukr.net
[3] Erwin Schrödinger International Institute for Mathematics and Physics, University of Vienna, 1090 Vienna, Austria
[4] Department of Electronic Properties of Materials, Faculty of Physics, University of Vienna, 1090 Wien, Austria
* Correspondence: viktor.schlosser@univie.ac.at; Tel.: +43-1-4277-72611

SUPPLEMENTARY INFORMATION

Supplementary Note S1, Elaborating interphase regions

The expressions for functions Φ_n (Equation (6) in the main text) were obtained using the relations given in Ref. [12]:

$$\Phi_0 = \Phi_0(\kappa_m, \kappa_C) = \frac{\kappa_m - \kappa_C}{\kappa_C + \frac{\kappa_m - \kappa_C}{3}}, \quad \text{(S1)}$$

$$\Phi_1 = \Phi_1(\alpha, \kappa_C) = \frac{1}{3}\left[\frac{2(FK_{11} - \kappa_C)}{\kappa_C + S_{11}(\alpha)(FK_{11} - \kappa_C)} + \frac{(FK_{33} - \kappa_C)}{\kappa_C + S_{33}(\alpha)(FK_{33} - \kappa_C)}\right], \quad \text{(S2)}$$

$$\Phi_2 = \Phi_2(\kappa_C) = \frac{1}{3}\frac{FK_2 - \kappa_C}{\kappa_C + \frac{FK_2 - \kappa_C}{3}}, \quad \text{(S3)}$$

$$\Phi_3 = \Phi_3(\alpha, \kappa_C) = \frac{1}{3}\left[\frac{2(\Phi K_{11} - \kappa_C)}{\kappa_C + S_{11}(\alpha)(\Phi K_{11} - \kappa_C)} + \frac{(\Phi K_{33} - \kappa_C)}{\kappa_C + S_{33}(\alpha)(\Phi K_{33} - \kappa_C)}\right], \quad \text{(S4)}$$

where $S_{ij}(\alpha)$ are the components of the 2-rank Eshelby tensor [S1]:

$$S_{11}(\alpha) = S_{22}(\alpha) = \frac{\alpha}{2(1-\alpha^2)^{3/2}}\left[\arccos(\alpha) - \alpha(1-\alpha^2)^{1/2}\right], \; if \; \alpha < 1 \quad \text{(S5)}$$

$$S_{11}(\alpha) = S_{22}(\alpha) = \frac{\alpha}{2(\alpha^2-1)^{3/2}}\left[\alpha(\alpha^2-1)^{1/2} - \operatorname{arccosh}(\alpha)\right], \; if \; \alpha > 1 \quad \text{(S6)}$$

$$S_{33} = 1 - S_{11}, \quad \text{(S7)}$$

where $\alpha = L_Z/L_X$ is the thickness-to-length aspect ratio of the spheroid-shaped graphene nanoplateletelets. κ_C is unknown thermal conductivity of the composite, and κ_m is intrinsic thermal conductivity of the epoxy matrix.

Functions FK_2 and FK_{nn} ($n = 1,2$) describe effective thermal conductivities of free (i.e. unassembled) particles surrounded by interphase polymer layers. For free anatase particles

$$\kappa_{2,eff} = FK_2 = FK_2(h_2, \kappa_2, r_2) = Kb_2(h_2, r_2) \cdot \left\{1 + \frac{[1 - \varphi_{i2}(h_2)] \cdot [\kappa_2 - Kb_2(h_2, r_2)]}{Kb_2(h_2, r_2) + \frac{1}{3}\varphi_{i2}(h_2) \cdot [\kappa_2 - Kb_2(h_2, r_2)]}\right\}, \quad \text{(S8)}$$

where κ_2 is the intrinsic thermal conductivity of anatase nanoparticles. Anatase-epoxy interphase layer is characterized by its thickness h_2, volume portion φ_{i2}, Kapitza thermal boundary resistance r_2 and thermal conductivity Kb_2

$$Kb_2(h_2, r_2) = \frac{h_2}{2r_2}, \tag{S9}$$

$$\varphi_{i2}(h_2) = 1 - \frac{R_2^3}{(R_2 + h_2)^3}, \tag{S10}$$

where R_2 is average radius of the particles and the factor 1/3 in the above equations is due to $S = 1/3$ for spherical particles. For free graphene nanoplatelets

$$FK_{nn} = FK_{nn}(\alpha, h_1, \kappa_{gnn}, r_1)$$
$$= Kb_1(h_1, r_1) \cdot \left\{1 + \frac{[1 - \varphi_{i1}(h_1)]\cdot[\kappa_{gnn} - Kb_1(h_1, r_1)]}{Kb_1(h_1, r_1) + \varphi_{i1}(h_1)\cdot S_{nn}(\alpha)\cdot[\kappa_{gnn} - Kb_1(h_1, r_1)]}\right\}, \tag{S11}$$

where $\kappa_{g11}, \kappa_{g22}$ and κ_{g33} are intrinsic in-plane ($n = 1,2$) and cross-plane ($n = 3$) thermal conductivities of graphene nanoplatelets.

Graphene-epoxy interphase layer is characterized by its thickness h_1, volume portion φ_{i1}, Kapitza thermal boundary resistance r_1 and thermal conductivity Kb_1

$$Kb_1(h_1, \kappa_{10}) = \frac{h_1}{2r_1}, \tag{S12}$$

$$\varphi_{i1}(h_1) = 1 - \frac{\left(\frac{L_Z}{2}\right)\cdot\left(\frac{L_X}{2}\right)^2}{\left(\frac{L_Z}{2}+h_1\right)\cdot\left(\frac{L_X}{2}+h_1\right)^2}, \tag{S13}$$

where $L_X = L_Y = 5$ μm and $L_Z = 50$ nm are average sizes of the graphene nanoplatelets in the X, Y and Z direrctions giving $\alpha = \frac{L_Z}{L_X} = 0.01$.

Finally, ΦK_{nn} are effective thermal conductivities of self-assembled (hybrid) graphene@anatase particles encapsulated in its interphase layer

$$\kappa_{1nn,eff} = \Phi K_{nn} = \Phi K_{nn}(\alpha, h_1, h_2, p_1, p_2, C_1, C_2, r_2, r_{21})$$
$$= Kb_2(h_2, r_2) \cdot \left\{1 + \frac{[1-\varphi_{i3}(h_1,h_2,p_1,p_2,C_1,C_2)]\cdot[F\kappa_{gnn}(\alpha,p_1,p_2,C_1,C_2,r_{21})-Kb_2(h_2,r_2)]}{Kb_2(h_2,r_2)+\varphi_{i3}(h_1,h_2,p_1,p_2,C_1,C_2)\cdot S_{nn}(\alpha)\cdot[F\kappa_{gnn}(\alpha,p_1,p_2,C_1,C_2,r_{21})-Kb_2(h_2,r_2)]}\right\}, \tag{S14}$$

where $F\kappa_{gnn}$ are effective thermal conductivities of anatase-covered graphene nanoplatelets ($F\kappa_{g11}$ – in-plane and $F\kappa_{g33}$ – cross-plane conductivities)

$$F\kappa_{gnn} = F\kappa_{gnn}(\alpha, p_1, p_2, C_1, C_2, r_{21})$$
$$= Kb_{21}(p_1, p_2, C_1, C_2, r_{21}) \cdot \left\{1 + \frac{[1-\varphi_{i30}(p_1,p_2,C_1,C_2)]\cdot[\kappa_{gnn}-Kb_{21}(p_1,p_2,C_1,C_2,r_{21})]}{Kb_{21}(p_1,p_2,C_1,C_2,r_{21})+\varphi_{i30}(p_1,p_2,C_1,C_2)\cdot S_{11}(\alpha)\cdot[\kappa_{gnn}-Kb_{21}(h_1,p_1,p_2,C_1,C_2,r_{21})]}\right\}, \tag{S15}$$

where $p_{1,2}$ are the mass portions of free (unassembled) filler's "1" (i.e. graphene) and "2" (i.e. anatase), respectively, and C_1 and C_2 are the mass concentrations of the fillers.

Here, anatase covering is characterized by effective thickness h_{21}

$$h_{21} = h_{21}(p_1, p_2, C_1, C_2) = \frac{1-p_2}{1-p_1} \cdot \frac{C_2}{C_1} \cdot \frac{\rho_1}{\rho_2} \cdot \frac{v_1}{s_1}, \tag{S16}$$

where s_1 and v_1 are the surface area and volume of the single graphene nanoplatelet.

Next, the thermal interaction between graphene and anatase covering is described by Kapitza thermal boundary resistance r_{21} and effective thermal conductivity Kb_{21}

$$Kb_{21}(p_1, p_2, C_1, C_2, r_{21}) = \frac{h_{21}(p_1,p_2,C_1,C_2)}{2r_{21}}. \tag{S17}$$

Also, φ_{i30} is the volume fraction of anatase in the hybrid particle

$$\varphi_{i30} = \varphi_{i30}(p_1, p_2, C_1, C_2)$$
$$= 1 - \frac{\left(\frac{L_Z}{2}\right)\cdot\left(\frac{L_X}{2}\right)^2}{\left[\frac{L_Z}{2}+h_{21}(p_1,p_2,C_1,C_2)\right]\cdot\left[\frac{L_X}{2}+h_{21}(p_1,p_2,C_1,C_2)\right]^2}, \quad \text{(S18)}$$

whereas φ_{i3} is the volume fraction of interphase polymer layer surrounded the hybrid particle:

$$\varphi_{i3} = \varphi_{i3}(h_1, h_2, p_1, p_2, C_1, C_2)$$
$$= 1 - \frac{\left[\frac{L_Z}{2}+h_{21}(p_1,p_2,C_1,C_2)\right]\cdot\left[\frac{L_X}{2}+h_{21}(p_1,p_2,C_1,C_2)\right]^2}{\left[\frac{L_Z}{2}+h_{21}(p_1,p_2,C_1,C_2)+h_2\right]\cdot\left[\frac{L_X}{2}+h_{21}(p_1,p_2,C_1,C_2)+h_2\right]^2}. \quad \text{(S19)}$$

The above expressions for effective thermal conductivities for graphene, anatase and hybrid particles have been written by using the Nan-Birringer-Clarke-Gleiter model [105].

In writing Equation S16, the underlying assumption is that anatase nanoparticles uniformly cover graphene nanoplatelets, thus forming a solid layer with effective thickness of h_{21}. The expression for h_{21} can easily be derived from such parameters as masses ($m_{1,2}$), volumes ($v_{1,2}$), surface areas ($s_{1,2}$) and mass densities ($\rho_{1,2}$) of the nanoparticles. Taking into account that

$$m_{1,2} = \rho_{1,2} \cdot v_{1,2},$$

the total number of anatase nanoparticles per single graphene nanoplatelet can be calculated as

$$N_{21}(p_1, p_2, C_1, C_2) = \frac{(1-p_2)\frac{C_2}{m_2}}{(1-p_1)\frac{C_1}{m_1}} = \frac{1-p_2}{1-p_1}\cdot\frac{C_2}{C_1}\cdot\frac{\rho_1}{\rho_2}\cdot\frac{v_1}{v_2}. \quad \text{(S20)}$$

Then, assuming the volume of the covering layer is $v_{21} = h_{21}s_1$, one can equate

$$N_{21}(p_1, p_2, C_1, C_2)\cdot v_2 = h_{21}(p_1, p_2, C_1, C_2)\cdot s_1. \quad \text{(S21)}$$

From Equation S21 we can obtain the relation to calculate h_{21}

$$h_{21}(p_1, p_2, C_1, C_2) = N_{21}(p_1, p_2, C_1, C_2)\cdot\frac{v_2}{s_1}. \quad \text{(S22)}$$

Inserting Equation S20 into Equation S22, one gets Equation S16.

If the nanoplatelet is considered as a rectangular parallelepiped, then its volume and surface area are given by

$$v_1 = v_{1,1} = L_X \cdot L_Y \cdot L_Z,$$
$$s_1 = s_{1,1} = 2(L_X \cdot L_Y + L_X \cdot L_Z + L_Y \cdot L_Z). \quad \text{(S23)}$$

When it has a shape of an oblate spheroid ($L_X = L_Y > L_Z$) one can find considering L_X, L_Y and L_Z as principal diameters [S2]:

$$v_1 = v_{1,2} = (\pi/6)\cdot L_X \cdot L_X \cdot L_Z$$
$$s_1 = s_{1,2} = (\pi/4)\cdot L_X^2 \cdot\left[1 + \frac{1-e^2}{e}\cdot \text{arctanh}\,(e)\right] \quad \text{(S24)}$$

where e is the spheroid's excentricity

$$e = \sqrt{1 - \alpha^2} = \sqrt{1 - \left(\frac{L_Z}{L_X}\right)^2}.$$

In our experiments $\alpha = 0.01$ and $e = 0.99995$.

Strictly speaking, Equation S21 is only accurate for a case of parallelepiped-shaped nanoplatelets. But when the nanoplatelet is spheroid-shaped, h_{21} should be evaluated by using the relation

$$N_{21}(p_1, p_2, C_1, C_2) \cdot v_2 \;=\; v_{1,2}(L_X + 2h_{21}, L_Y + 2h_{21}, L_Z + 2h_{21}) \;-\; v_{1,2}(L_X, L_Y, L_Z) \quad \text{(S25)}$$

where $v_{1,2}$ is determined by Equations S23 and S24.

In order to describe the case when there is no interphase interaction between the fillers, one should put $p_1 = p_2 = 1$ using Equations S26 and S27 instead of Equations S2and S3:

$$\Phi_{10} \;=\; \Phi_{10}(\alpha, \kappa_{g11}, \kappa_{g33}, \kappa_C) \;=\; \frac{1}{3}\left[\frac{2(\kappa_{g11} - \kappa_C)}{\kappa_C + S_{11}(\alpha)(\kappa_{g11} - \kappa_C)} + \frac{(\kappa_{g33} - \kappa_C)}{k_C + S_{33}(\alpha)(\kappa_{g33} - \kappa_C)}\right], \quad \text{(S26)}$$

$$\Phi_{20} \;=\; \Phi_2(\kappa_2, \kappa_C) \;=\; \frac{1}{3}\frac{\kappa_2 - \kappa_C}{k_C + \frac{\kappa_2 - \kappa_C}{3}}\,. \quad \text{(S27)}$$

Treating mechanical properties of hybrid graphene@anatase nanosheets: Soft- and hard-sphere approximations

Different architectures of hybrid MLG@TiO_2 nanosheets configured in a sandwich-like structure are shown in Figure 4 (main text). Using the data given in Table 1 (main text), one can easily evaluate surface areas ($s_{1,2}$) volumes ($v_{1,2}$) and masses ($m_{1,2}$) of the filling particles. Numerical calculations give $s_{1,1}$ = 5.10×10^{-11} m^2, $v_{1,1}$ = 1.25×10^{-18} m^3, and $v_{1,1}/s_{1,1}$ = 24.51 nm (for parallelepiped-shaped nanoplatelets); $s_{1,2} \approx$ 3.929×10^{-11} m^2, $v_{1,2} \approx$ 6.55×10^{-23} m^3, and $v_{1,2}/s_{1,2} \approx$ 16.66 nm (for spheroid-shaped nanoplatelets); s_2 = $4\pi R_2^2 \sim$ 7.85×10^{-15} m^2, $v_2 = \frac{4}{3}\pi R_2^3 \sim$ 8.18×10^{-24} m^3; $m_{1,1} \;=\; \rho_{f,1} \cdot v_{1,1} \sim$ 2.83×10^{-12} g (parallelepiped), $\rho_{f,1} \cdot v_{1,2} \sim$ 1.48×10^{-12} g (spheroid), $m_2 \;=\; \rho_{f,2} \cdot v_2 \sim$ 2.54×10^{-16} g. Here, we take $L_X = L_Y$ = 5.0×10^3 nm and L_Z = 50.0 nm as average values for nanoplatelete's sizes evaluated from the SEM-images [14].

These calculations show that the thickness h_{21} of the layer covering a spheroid will be as much as 24.51/16.66 ≈ 1.47 times less than that on a parallelepiped, provided that other conditions remain the same. The difference in the volume-to-surface area ratio between a parallelepiped and an spheroid is due to that the both surface area and volume decrease ($s_{1,2}/s_{1,1}$ ≈ 0.7704, $v_{1,2}/v_{1,1}$ ≈ 0.5236) then a parallelepiped is transformed into an oblate spheroid of the same linear dimensions. Also, when $p_1 = p_2 = p$ and $C_1 = C_2 = C$, one finds from Equation S20 that $N_{21}(p, p, C, C) = N_{21,1} \approx$ 11149 (for parallelepiped-shaped nanoplatelets) or $N_{21}(p, p, C, C) = N_{21,2} \approx 5838$ (for spheroid-shaped nanoplatelets).

We note that the development of the concept of "the effective thickness" follows a somewhat tortuous path. Below we give a brief explanation of this issue within the context of our approach. As mentioned above, Equation S21 implies that anatase nanoparticles form a uniform solid layer on the surface of a nanoplatelet. In other words, the TiO_2 nanoparticles are treated as "soft spheres" capable of spreading out over the surface of graphene nanoplatelet like a viscid oil and thus forming a uniform solid covering. In the opposite case of "hard spheres", Equation S21 is not valid and an estimate of the effective layer thickness needs further clarification and is hereafter referred to as $H_{21,eff}$. From a practical point of view, it is important to predict permissible variation ranges for mass concentrations C_1 and C_2, and related values of volume concentrations $\varphi_n(C_1, C_2)$ (see Supplementary Note S2) where the assembling effect can be effectively used for tailoring composite's thermal properties.

Simplistically, assembling should be significant on the condition that TiO_2 does not fully cover the surface of MLGs. The largest possible values of the mass concentrations can then be directly evaluated. In the hard-sphere approximation, the maximal allowed number (N_0) of the TiO_2 nanoparticles with the radius of R_2 which can be packed tightly over a flat or curved MLG surface having the area of s_1 is given by

$$N_0 = \frac{s_1}{4R_2^2}. \tag{S28}$$

Numerical calculations give $N_0 = N_{01} = 2.0400\times10^4$ (or $N_0 = N_{02} = 1.5716\times10^4$) for parallelepiped-shaped (or spheroid-shaped) nanoplatelets, so that $N_{02}/N_{01} \approx 0.7704$. These N_0 particles cover (or, speaking somewhat loosely, "shade") the area of s_{10}

$$s_{10} = N_0 \cdot \pi R_2^2 = \frac{\pi}{4} s_1.$$

Thus, when the condition

$$N_{21}(p_1, p_2, C_1, C_2) = N_0 \tag{S29}$$

is satisfied, $H_{21,eff}$ is equal to $2R_2 = 50$ nm and the portion Δs of the surface area s_1

$$s_1 - s_{10} = \left(1 - \frac{\pi}{4}\right) s_1 \approx 0.21 s_1$$

remains uncovered ("unshaded"). For the comparison, when Equation S29 is met, h_{21} can be evaluated as $h_{21,0}$ from

$$\frac{4\pi}{3} R_2^3 \cdot N_0 = s_1 \cdot h_{21,0}$$

as

$$h_{21,0} = \frac{\pi}{3} R_2 \approx 26.18 \text{ nm}$$

independent of the way of modeling nanoplatelet's shape, i.e. $h_{21,0} < 2R_2$.

When $N_{21}(p_1, p_2, C_1, C_2) > N_0$, it may be assumed that the ensemble of $N_{21}(p_1, p_2, C_1, C_2)$ spherical particles with radii R_2 can build NL_2 layers given by

$$NL_2(p_1, p_2, C_1, C_2) = \frac{N_{21}(p_1, p_2, C_1, C_2)}{N_0}. \tag{S30}$$

The effective thickness $H_{21,eff}$ of the tightly-packed covering can amount to

$$\begin{aligned} H_{21,eff}(p_1, p_2, C_1, C_2) &= 2R_2 + (2R_2 - \delta_2) \cdot [NL_2(p_1, p_2, C_1, C_2) - 1] \\ &= 2R_2 \cdot NL_2(p_1, p_2, C_1, C_2) - \delta_2 \cdot [NL_2(p_1, p_2, C_1, C_2) - 1], \end{aligned} \tag{S31}$$

where δ_2 characterizes a lowering of the layer's thickness ($2R_2$) due to the effect of tight packing of spherical objects as shown in Figure 4(**a**). From the geometrical considerations, one can easily find

$$\delta_2 = 2\left(-\frac{\sqrt{3}}{2}\right) R_2 \approx 6.7 \text{ nm}$$

and algebraic transformations give

$$2R_2 NL_2(p_1, p_2, C_1, C_2) = \frac{N_{21}(p_1, p_2, C_1, C_2)}{s_1} \cdot 8R_2^3 = \frac{6}{\pi} N_{21}(p_1, p_2, C_1, C_2) \frac{v_2}{s_1} = \frac{6}{\pi} \cdot h_{21}(p_1, p_2, C_1, C_2), \tag{S32}$$

where $v_2 = \frac{4\pi}{3} R_2^3$ is the volume of anatase spherical particle of the radius R_2. Thus, one gets

$$H_{21,eff}(p_1, p_2, C_1, C_2) = \frac{6}{\pi} \cdot h_{21}(p_1, p_2, C_1, C_2) - \delta_2 \cdot [NL_2(p_1, p_2, C_1, C_2) - 1]. \tag{S33}$$

In our experiments, two kind of nanocomposite samples have been used, which differ from each other by the ratio of C_1/C_2:

$(C_1, C_2) = (0.01, 0.01)$ and $(C_1, C_2) = (0.01, 0.05)$. In the case of $p_1 = p_2 = p$, numerical calculations give:

for «soft spheres» by using Equation S21

$h_{21}(p, p, 0.01, 0.01) \approx 14.3$ nm, $h_{21}(p, p, 0.01, 0.05) \approx 71.5$ nm (parallelepiped-shaped nanoplatelets),

$h_{21}(p, p, 0.01, 0.01) \approx 9.7$ nm, $h_{21}(p, p, 0.01, 0.05) \approx 48.6$ nm (spheroid-shaped nanoplatelets);

for «hard spheres», by using Equation S33

$H_{21,eff}(p, p, 0.01, 0.01) \approx 30.4$ nm, $H_{21,eff}(p, p, 0.01, 0.05) \approx 125.0$ nm (parallelepiped-shaped nanoplatelets),

$H_{21,eff}(p, p, 0.01, 0.01) \approx 22.8$ nm, $H_{21,eff}(p, p, 0.01, 0.05) \approx 87.1$ nm (spheroid-shaped nanoplatelets).

Loading dependences of the effective thickness calculated in the soft-sphere approximation for different geometrical configurations of the nanoplatelets are shown in Figure S1. A noticeable difference between curves 1 and 2 is observed, which are obtained with the parallelepiped- and spheroid-shaped graphene nanoplatelets, respectively. The behavior predicted from the exact solutions (curve 3) provides a noticeably good agreement with the approximate results (curve 1) showing that the approximations are rather accurate for practical purposes of finding κ_C by using the approximate Equations S21 and S24 instead of the exact Equations S25 and S24.

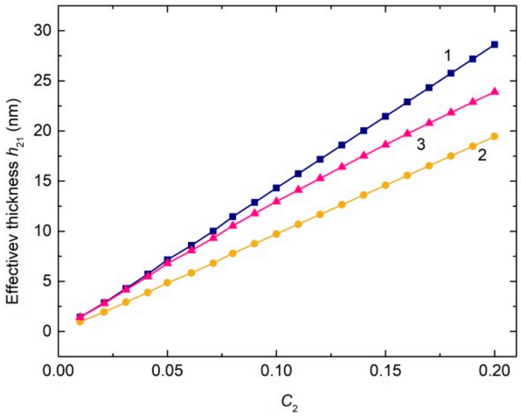

Figure S1. Loading dependences of the effective thickness (h_{21}) of anatase layer on graphene nanoplatelets calculated in the soft-sphere approximation for different geometrical configurations of the MLGs: 1 – parallelepiped-shaped MLGs (approximate evaluation by using Equations S25 and S23), 2 – spheroid-shaped MLGs (approximate evaluation by using Equations S25 and S24), 3 – spheroid-shaped MLGs (exact evaluation by using Equations S25 and S24).

Finally, using Equations S20 and S29, one can find the ratio $r = C_1/C_2$, when the number N_0 can be reached or the condition $NL_2 = 1$ is satisfied. Supposing $p_1 = p_2$ gives $r = r_0 = (C_1/C_2)_0 = N_0(m_2/m_1) \approx$ 1.83 or 2.69 for parallelepiped-like or spheroid-like nanoplatelets, respectively. From the other hand, for arbitrary values of (C_1, C_2) at $p_1 = p_2 = p$, the covered portion of the platelet's surface is

$$\frac{\pi R_2^2 N_{21}(p, p, C_1, C_2)}{s_{1,1}} \approx 0.4292 \cdot \frac{C_2}{C_1}$$

for parallelepiped-like nanoplatelets or

$$\frac{\pi R_2^2 N_{21}(p,p,C_1,C_2)}{s_{1,2}} \approx 0.2917 \cdot \frac{C_2}{C_1}$$

for spheroid-like nanoplatelets. These estimations help one to understand that the concentration set of $C_1 = 0.01$ and $C_2 = 0.05$ have been chosen to ensure entire covering of the graphene nanoplatelets with anatase nanoparticles.

In fact, the condition $NL_2 = 1$ defines a rough upper bound on the values of C_1 and C_2 as the probability to obtain more layers of anatase on graphene nanoplatelets by using a self-assembling techniques is extremely low. Another important bounds for choosing C_1 and C_2 come from the effects of interphase layer overlapping and percolation thresholds (Section 3.4 in the main text).

Some of the computed parameters for MLG@TiO_2 hybrid nanoparticles are given in Table S1, which includes the numbers of graphene nanoplatelets ($N_{1,1}$ and $N_{1,2}$ for the parallelepiped- and spheroid-shaped configurations, respectively) and anatase nanoparticles (N_2) per unit mass (1 g) of the nanocomposite, their ratio $N_{21} = N_2/N_1$, the number NL_2 of the anatase layers in "the hard-sphere" approximation and the effective thicknesses of anatase nanoparticles covered the graphene nanoplatelets evaluated in the "soft-sphere" ($h_{21,eff}$) and "hard-speres" ($H_{21,eff}$) approximations.

Table S1. Numbers of the MLG (N_1) and TiO_2 (N_2) nanoparticles, their ratio (N_{21}), number of anatase layers (NL_2) in "the hard-sphere" approximation, and the effective thicknesses of anatase nanoparticles covered the graphene nanoplatelets, evaluated in "soft-spere" ($h_{21,eff}$) and "hard-speres" ($H_{21,eff}$) approximations.

C_1/C_2	$N_{1,1}/N_{1,2}$	N_2	N_{21}	$h_{21,eff}$ (nm)	NL_2	$H_{21,eff}$ (nm)
0.01:0.01	3.532×10^{8}	3.938×10^{12}	11149	14.3	0.55	–
	6.746×10^{8}	3.938×10^{12}	5838	9.7	0.09	–
0.01:0.05	3.532×10^{8}	1.9689×10^{14}	55745	71.5	2.73	130.8
	6.746×10^{8}	1.9689×10^{14}	29188	48.6	0.46	90.0
0.05:0.01	1.766×10^{10}	3.938×10^{12}	2230	2.86	0.11	–
	3.373×10^{10}	3.938×10^{12}	1167	1.94	0.02	–
0.05:0.05	1.766×10^{10}	1.9689×10^{14}	11149	14.3	0.55	–
	3.373×10^{10}	1.9689×10^{14}	5838	9.7	0.09	–

First, in contrast to the hard-sphere approximation, the soft-sphere approach suggests solid anatase layer, which completely covers the graphene nanoplatelets and may be very thin with the effective

thickness going to zero when $C_2 \to 0$. Obviously, this clear distinction made between the two approximations is blurred out as R_2 decreases.

Second, the numerical estimates made allow us to understand why the anatase layer thickness determined by Equation S21 was named as an "effective" thickness. This is because the anatase nanoparticles are considered as being absolutely compliant and, as a consequence, Equation S21 imposes no restrictions on the thickness, whereas real nanoparticles are hard and the thickness cannot be less than the particle's diameter. However, the soft-sphere approximation simplifies essentially a quantitative description of interphase layer around the hybrid (two-layered) nanosheet by using the above equations.

As far as we know from the literature, the problem of describing two-layered nanostructures in a case of non-solid outer layer has not yet been analyzed. The main difficulty being that the contacts between the layers are dotty and phonon transport through the contacts is governed by the ballistic mechanisms. The above soft-spheres approach and related concept of the effective thickness (see Equation S16) allow to simplify simulation by replacing a dotty interface by continuous one and describing interface thermal transport in the hybrid anatase@graphene nanosheets by the phenomenological thermal boundary resistance r_{21} (see Equation S17).

As stated in the main text, an average value r_{1av} of r_1 can be given the form

$$\frac{1}{r_{1av}} = \frac{\frac{L_X L_Z}{r_{1X}} + \frac{L_Y L_Z}{r_{1Y}} + \frac{L_X L_Y}{r_{1Z}}}{L_X L_Z + L_Y L_Z + L_X L_Y}.$$

Putting $r_{1X} = r_{1Y}$, $L_Z = 50$ nm, $L_X = L_Y = 5\times10^3$ nm (average linear dimensions of our nanoplatelets) yields

$$\frac{1}{r_{1av}} = \frac{1.96 \times 10^{-2}}{r_{1X}} + \frac{0.9804}{r_{1Z}},$$

$$\frac{1}{r_{1av}} = \frac{r_{1Z}}{0.9804 + 1.96 \times 10^{-2} \frac{r_{1Z}}{r_{1X}}}.$$

Numerical values of r_{1X} and r_{1Z} have been theoretically calculated in Ref. [S3] for copper-MLG interfaces. It was found that the copper and cross-plane multilayer graphene ($n = 8$) nanocomposite has a thermal interface conductance of 0.84×10^8 W m^{-2} K^{-1} while the copper and in-plane multilayer graphene ($n = 8$) nanocomposite shows a larger thermal interface conductance of 2.30×10^8 W·m^{-2} K^{-1}.

Equating $\frac{r_{1Z}}{r_{1X}} = \frac{1}{84} \times 230 \approx 2.738$ readily gives $r_1 \approx 1.0144 \cdot r_{1Z}$. Thus, approximating $r_1 \approx r_{1X} = r_{1Z}$ would result in the negligible error in the calculation of the thermal conductivity κ_C of graphene-epoxy nanocomposites.

It is quite appropriate to compare the computational accuracy of both approximations proposed above. First, it should be noted that the soft-spheres approximation (SSA) implies that the Kapitza thermal boundary resistance of MLG@TiO_2-hybrid-epoxy interface is r_2. A transition to the hard-spheres approximations (HSA) requires r_2 to be substituted by r_h which is given by

$$r_h = r_h(p_1, p_2, C_1, C_2, R_2) \approx \frac{s_1 r_{1av} r_2}{[s_1 - s_{21}(p_1, p_2, C_1, C_2, R_2)] r_2 + s_{21}(p_1, p_2, C_1, C_2) r_1}$$

$$= \frac{r_2}{\frac{s_{21}(p_1, p_2, C_1, C_2, R_2)}{s_1} + \left[1 - \frac{s_{21}(p_1, p_2, C_1, C_2, R_2)}{s_1}\right] \frac{r_2}{r_{1av}}}, \quad \text{(S34)}$$

where $s_{21}(p_1, p_2, C_1, C_2, R_2)$ is the portion of graphene's surface area covered by "hard" spherical anatase particles:

$$s_{21}(p_1, p_2, C_1, C_2, R_2) = \pi R_2^2 N_{21}(p_1, p_2, C_1, C_2).$$

Putting R_2 = 25 nm, $p_1 = p_2$, and $C_1 = C_2 = 0.01$, we obtain $s_{21}/s_1 \approx 0.429$ for parallelepiped-shaped nanoplatelets. When putting r_1=3.5·10^{-9} W^{-1}·m^{2}·K^{-1} in Equation S34 one obtains $r_h/r_2 \approx$ 0.4855 (or ≈ 0.316) for r_2 = 10^{-8} (or 10^{-9}) W^{-1}·m^{2}·K^{-1}.

As a consequence of the condition $r_h < r_2$, values of κ_C evaluated in HSA will be higher than those evaluated in SSA. However, the increment in κ_C is rather small. For example, putting α = 0.01 and $h_1 = h_2$ = 24 nm yields the enhancement in κ_C varied in the range from 0.3 to 1.6% for r_2 = 10^{-8} W^{-1}·m^{2}·K^{-1} and 0.23 to 0.37% for r_2 = 10^{-9} W^{-1}·m^{2}·K^{-1}, when r_{21} varies in range from 5·10^{-7} to 5·10^{-6} W^{-1}·m^{2}·K^{-1}.

The difference in evaluating κ_C in HAS and SSA appears to decrease upon increasing the ratio C_2/C_1 or r_h. The bound value of r_h is reached when condition S29 is met (at the complete covering of graphene's surface with anatase) and $s_{21}/s_1 \approx 0.79$. If C_2/C_1 increases further, certain portions of both anatase and graphene fillers remain unassembled. For that cases, HSA becomes more appropriate for κ_C calculations. However, the calculations become somewhat cumbersome since both parameters p_1 and p_2 cannot be considered independent variables anymore, and their dependence on C_1 and C_2 should be taken into account.

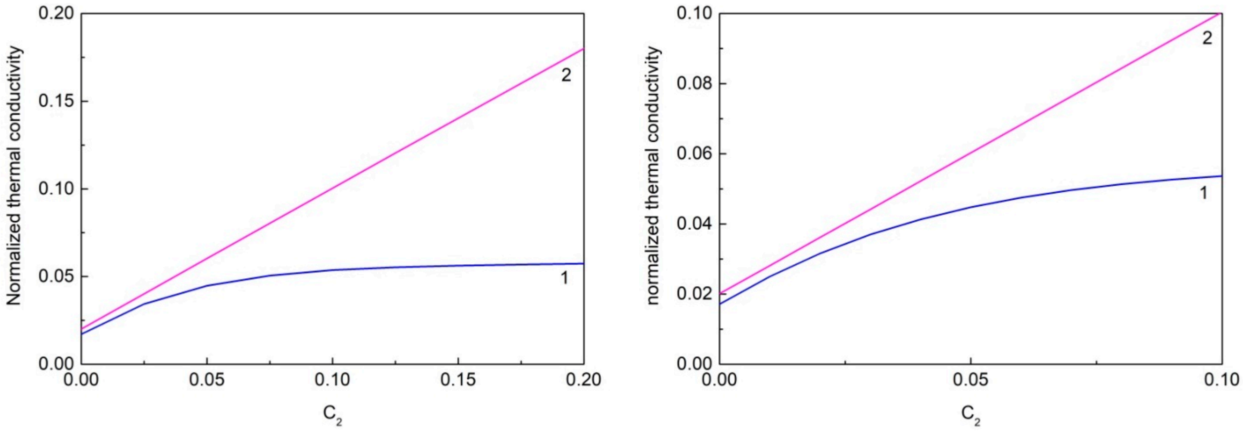

(**a**) (**b**)

Figure S2. Calculated loading dependence of normalized effective in-plane $\kappa_{g11,eff}/\kappa_{g11}$ (curves 1) and out-of-plane $\kappa_{g33,eff}/\kappa_{g33}$ (curves 2) thermal conductivities of hybrid MLG@TiO_2 nanoparticles at κ_{g11} = 600.0 W m^{-1} K^{-1} and κ_{g33} = 6.0 W m^{-1} K^{-1}. Calculations were performed by using Equation S14.

Finally, to understand the reason for increasing κ_C with increasing C_2, the loading dependencies of the filler thermal conductivities have been evaluated and plotted in Figure S2. It shows calculated loading

dependencies of the effective thermal conductivities $\kappa_{g11,eff}$ (long-range plot) and $\kappa_{g33,eff}$ (short-range plot) normalized by the corresponding intrinsic values κ_{g11} and κ_{g33} respectively.

It can be seen that $\kappa_{g33,eff}/\kappa_{g33}$ increases much faster than $\kappa_{g11,eff}/\kappa_{g11}$ with increasing C_2. Therefore, the increase in κ_C with increasing C_2 observed in Figure 5 is obviously due to increased $\kappa_{g33,eff}$, which in turn is due to increased $h = h_{21}$ of the anatase layer covering graphene nanoplatelets (see Table S1). The facts that $\kappa_{g11,eff}(C_2 = 0) \ll \kappa_{g11}$ and $\kappa_{g33,eff}(C_2 = 0) \ll \kappa_{g33}$ are furthermore expected to originate from the graphene-epoxy Kapitza thermal boundary resistance and related interphase layers.

Supplementary Note S2, Volume concentrations of the constituents

Starting from the general equations for mass and volume balances for a three-filling-phase composite of the volume V_C and mass M_C and taking related interphase region into account,

$$\sum_n M_n = M_C , \tag{S35}$$

$$\sum_n V_n = V_C , \tag{S36}$$

together with the density relations,

$$M_{n,C} = \rho_{n,C} V_{n,C} , \tag{S37}$$

one can obtain, after some tortuous algebra, the expressions to calculate the composite's density ρ_C and volume concentrations of the constituents $\varphi_n = V_n/V_C$ ($n = 0, \ldots, 5$) (Equation (5) in the main text) via the mass concentrations of the staple fillers ($n = 1, 2$) $C_{1,2} = M_{1,2}/M_C$:

$$\rho_C = \left[\frac{1-C_1-C_2-p_1F_1\frac{\rho_4}{\rho_1}C_1-p_2F_2\frac{\rho_5}{\rho_2}C_2-(1-p_2)F_3\frac{\rho_5}{\rho_3}}{\rho_m} + \frac{C_1}{\rho_1} + \frac{C_2}{\rho_2} + p_1F_1\frac{C_1}{\rho_1} + p_2F_2\frac{C_2}{\rho_2} - (1-p_2)F_3\frac{C_2}{\rho_3}\right]^{-1} , \tag{S38}$$

$$\varphi_0 = \left(\frac{\rho_C}{\rho_0}\right) \cdot \left[1 - C_1 - C_2 - p_1F_1\frac{\rho_4}{\rho_1}C_1 - p_2F_2\frac{\rho_5}{\rho_2}C_2 - (1-p_2)F_3\frac{\rho_5}{\rho_3}\right] , \tag{S39}$$

$$\varphi_1 = p_1 \cdot \left(\frac{\rho_C}{\rho_1}\right) \cdot C_1 , \tag{S40}$$

$$\varphi_2 = p_2 \cdot \left(\frac{\rho_C}{\rho_2}\right) \cdot C_2 , \tag{S41}$$

$$\varphi_3 = (1-p_1) \cdot \left(\frac{\rho_C}{\rho_1}\right) \cdot C_1 + (1-p_2) \cdot \left(\frac{\rho_C}{\rho_2}\right) \cdot C_1 , \tag{S42}$$

$$\varphi_4 = p_1F_1\frac{\rho_C}{\rho_1}C_1 , \tag{S43}$$

$$\varphi_5 = p_2F_2\frac{\rho_C}{\rho_2}C_2 , \tag{S44}$$

$$\varphi_6 = (1-p_2)F_3\frac{\rho_C}{\rho_3}C_2 . \tag{S45}$$

Here, the subscript "$n = 0$" corresponds to a host matrix, "1" – to graphene nanoplatelets, "2" – to anatase nanoparticles, "3" – to self-assembled hybrid phase composed of fillers "1" and "2", and "4" and "5" - to interphase regions "$i1$" and "$i2$" surrounding the fillers. With this in mind, the interphase layer around the hybrid particles may be either "$i1$" or "$i2$", depending on what filler is being used as outer layer of the hybrid. The "$i2$" case is detailed here. The density of the hybrid particle is given by

$$\rho_3 = \frac{[(1-p_1)\cdot C_1 + (1-p_2)\cdot C_2]\cdot \rho_1 \cdot \rho_2}{(1-p_1)\cdot \rho_2 \cdot C_1 + (1-p_2)\cdot \rho_1 \cdot C_2} . \tag{S46}$$

In the above equations, $F_{1,2,3}$ are the volume factors which determine the ratio of the interphase layer's volume to the particle's volume. Assuming that the graphene nanoplatelets are ellipsoids having the axes L_X, L_Y and L_Z, and the anatase particles are spheres of radius R_2, we find

$$F_1 = \left(1+\frac{2h_{i1}}{L_X}\right)\cdot\left(1+\frac{2h_{i1}}{L_Y}\right)\cdot\left(1+\frac{2h_{i1}}{L_Z}\right)-1 \quad (S47)$$

$$F_2 = \left(1+\frac{h_{i2}}{R_2}\right)^3-1 \quad (S48)$$

$$F_3 = \left(1+\frac{2h_{i1}}{L_X+h_{21}}\right)\cdot\left(1+\frac{2h_{i1}}{L_Y+h_{21}}\right)\cdot\left(1+\frac{2h_{i1}}{L_Z+h_{21}}\right)-1 \quad (S49)$$

where h_{21} is the thikness of anatase layer covering graphene's nanoplatele (see below), $p_{1,2}$ are the mass portion of free (unassembled) fillers "1" and "2", respectively.

Supplementary Note S3, Thermal conductivity upon varying graphene thickness

Calculations are performed at κ_{g11} = 600.0 W m^{-1} K^{-1}, κ_{g33} = 6.0 W m^{-1} K^{-1}, $H_1 = H_2$ = 24 nm, r_{1X} = 1.28×10^{-9} m^2 K W^{-1}, r_{1Z} = 3.5×10^{-9} m^2 K W^{-1}, r_2 = 1.0×10^{-9} m^2 K W^{-1}, r_{21} = 2.0×10^{-10} m^2 K W^{-1} (at C_1 = 0.010, C_2 = 0.050), r_{21} = 1.456×10^{-10} m^2 K W^{-1} (at C_1 = 0.010, C_2 = 0.010) (complete coverage of the surface of MLGs with TiO_2).

Table S2. Variation of the computed thermal conductivity κ_C upon varying MLG thickness L_Z when keeping $L_X = L_Y$ = 5.0×10^{-6} m.

Thickness L_Z (nm)	$\alpha = L_X/L_Z$	κ_C (W·m^{-1}·K^{-1}) C_1 = 0.010, C_2 = 0.010	κ_C (W·m^{-1}·K^{-1}) C_1 = 0.010, C_2 = 0.050
10.0	0.002	0.378	0.328
25.0	0.005	0.330	0.331
50.0	0.01	0.286	0.313
100.0	0.02	0.250	0.283
250.0	0.05	0.222	0.245
500.0	0.1	0.212	0.226

Table S3. Variation of the computed thermal conductivity κ_C upon varying MLG thickness L_Z when keeping α = 0.01.

Thickness L_Z (nm)	$L_X = L_Y = \alpha L_Z$ (μm)	κ_C (W·m^{-1}·K^{-1}) C_1 = 0.010, C_2 = 0.010	κ_C (W·m^{-1}·K^{-1}) C_1 = 0.010, C_2 = 0.050
10.0	1.0	0.310	0.351
25.0	2.5	0.299	0.330
50.0	5.0	0.286	0.313
100.0	10.0	0.269	0.292
250.0	25.0	0.243	0.258
500.0	50.0	0.224	0.230

References

[S1] Wang, Y.; Shan, J. W.; Weng, G. J. Percolation threshold and electrical conductivity of graphene based nanocomposites with filler agglomeration and interfacial tunneling. *J. Appl. Phys.* **2015**, *118*, 06161. DOI: 10.1063/1.4928293

[S2] Soltani, A. Equations of spheroid and ellipsoid surface area by analytical method. *Cambridge Open Engage* 2022. Available online: https://www.cambridge.org/engage/coe/article-details/61f7a44963acba4ce3201236 (accessed on 24 October 2023).

[S3] Chang, S.-W.; Nair, A. K.; Buehler, M. J. Geometry and temperature effects of the interfacial thermal conductance in copper– and nickel–graphene nanocomposites. *J. Phys. Cond. Matter* **2012**, *24*, 245301. doi:10.1088/0953-8984/24/24/245301
